# Treatment of Acute Wounds With Recombinant Human-Like Collagen and Recombinant Human-Like Fibronectin in C57BL/6 Mice Individually or in Combination

**DOI:** 10.3389/fbioe.2022.908585

**Published:** 2022-05-19

**Authors:** Yunqing Dong, Weidong Zhu, Xiaoxuan Lei, Xin Luo, Qi Xiang, Xuanru Zhu, Qiao Pan, Panshi Jin, Biao Cheng

**Affiliations:** ^1^ The First School of Clinical Medicine, Southern Medical University, Guangzhou, China; ^2^ Department of Burn and Plastic Surgery, General Hospital of Southern Theater Command of PLA, Guangzhou, China; ^3^ Department of Oral and Maxillofacial Surgery/Pathology, Amsterdam UMC and Academic Center for Dentistry Amsterdam (ACTA), Vrije Universiteit Amsterdam, Amsterdam Movement Science, Amsterdam, Netherlands; ^4^ Institute of Biomedicine and Guangdong Provincial Key Laboratory of Bioengineering Medicine, Jinan University, Guangzhou, China

**Keywords:** recombinant human-like collagen (RHC), recombinant human-like fibronectin (rhFN), wound healing, fibroblast cell, re-epithelialization, collagen deposition, inflammation, angiogenesis

## Abstract

Wound repair is accomplished by the interaction between the cells involved in the repair and the extracellular matrix (ECM). Collagen is the main component of ECM, which is involved in transduction of signal, transportation of growth factors and cytokines. Fibronectin (FN) is also an important ECM, which participates in the initiation of fibroblast cell (FC) and promotes adhesion, migration, proliferation and differentiation of target cells. Compared with natural protein, the recombinant protein prepared by artificial method has the advantages of poor immunogenicity, wide range of sources, low cost and high activity. In this study, we used recombinant human-like collagen (RHC) and recombinant human-like fibronectin (rhFN) to treat acute wounds in C57BL/6 mice individually or in combination, and explored their effects on wound healing. Our study confirmed that these two recombinant proteins could effectively promote the proliferation, migration and adhesion of FCs. Meanwhile, it could positively regulate the healing speed and quality of acute wounds, re-epithelialization, collagen deposition, inflammation and angiogenesis. Moreover, we proved that the combination of the two was better than the treatment alone. Consequently, it has a good prospect as a new tissue material in the field of skin repair.

## 1 Introduction

Acute skin injury is very common, with high incidence rate and many complications. The prognosis is affected by many factors, such as the location, depth and area of the wound. If the acute skin injury can not be repaired in an orderly and timely manner, it may turn into a chronic refractory wound. Acute skin wound repair is a physiological process involving inflammatory cells, repair cells, ECM and a variety of growth factors, and ECM plays an important role in skin wound healing (Gurtner et al., 2008). Synthetic ECM and its key components are promising new materials for wound repair and tissue regeneration. ECM is the basis of extracellular microenvironment, which plays an important role in regulating biological behaviors such as proliferation, migration, adhesion, differentiation and apoptosis of cells, and can promote re-epithelialization, collagen deposition as well as regulate angiogenesis (Theocharis et al., 2016).

ECM is a complex three-dimensional network structure, which is composed of gel matrix and fibrous protein components. Fibrous proteins mainly contain collagen and elastin with structural support, as well as fibronectin (FN) and laminin (LN), which play a role in adhesion (Järveläinen et al., 2009). Collagen is not only the main component of ECM, but also the most abundant protein in human body. Most collagen can form structural scaffolds for wound healing and have good biocompatibility and degradability. It promotes tissue repair by promoting adhesion, chemotaxis and migration of cells (Colby and Prusiner, 2011). FN is also an important part of ECM. It can be used as a scaffold for cell migration, assist FCs to migrate to the wound, as well as promote collagen deposition and restore tissue structure (Patten and Wang, 2020). Previous studies have shown that collagen and FN are widely involved in all stages of wound repair (Mathew et al., 2021).

The wound healing process is complex regulated by many key factors, and collagen is widely involved in all stages of wound repair. In general, collagen transformation is slow, but it is accelerated during the remodeling and healing of skin injury (Jansen et al., 2018). In the early stage, collagen degradation fragments can recruit immune cells, such as monocytes-macrophages, to clear necrotic tissue and microorganisms (Yeung and Kelly, 2021). At the same time, in this primary stage, thrombin or collagen can release FN in platelets and help platelets bind firmly to fibrin and collagen in the form of covalent bonds to form thrombosis and cell matrix structures (Mosesson and Umfleet, 1970; Grinnell et al., 1981), which will contribute to the transition to the next phase. In the inflammatory stage, collagen will produce a strong and rapid inflammatory response, which is temporary and will subside soon, so as to promote wound healing. In the proliferative phase, on the one hand, collagen plays a chemotactic role, which significantly promotes the adhesion and effectively increases the proliferation of FN around the wound (Huang et al., 2012; Smithmyer et al., 2014; Lai et al., 2020). On the other hand, it can activate macrophages, induce the expression of various cytokines and growth factors, and promote angiogenesis (Lin et al., 2006; To and Midwood, 2011; Elbialy et al., 2020). Type I collagen can effectively stimulate angiogenesis *in vitro* and *in vivo* by recruiting endothelial cells to bind to specific integrin receptors. In addition, collagen can promote migration, re-epithelialization and collagen deposition of keratinocytes (Dang et al., 2015; Pal et al., 2016; Chen et al., 2019). At the same time, FN is mainly produced by FCs and macrophages in proliferative phase, which promotes the formation of granulation tissue, attracts endothelial cells and epithelial cells, promotes adhesion and migration of cells to matrix, and accelerates re-epithelialization (Cheng et al., 1988). Finally, in the process of wound remodeling, collagen, as a structural component of ECM, not only regulates the stable release of growth factors, intercellular adhesion and signal transduction, but also contributes to the elasticity of skin.

FN runs through all stages of wound repair. It can be used as a scaffold to recruit target cells to migrate to the wound and restore the tissue structure. Firstly, in the hemostatic period, platelets themselves do not participate in the agglutination of fibrinogen. FN derived plasma plays an important role in the accumulation and mutual adhesion of platelets, and firmly binds with fibrin and collagen in covalent form to form insoluble gel condensate (Eisinger et al., 2018). Next, in the middle stage of wound healing, FN gathers into a complex three-dimensional structure on the cell surface to regulate adhesion, migration, proliferation and apoptosis of cells in the process of skin wound healing. At the same time, collagen further forms the grid structure, which depends on the initial FN structure. In addition, as a non-specific opsonin, FN can mobilize and promote the phagocytic system to remove foreign bodies and purify the wound. Finally, in the stage of remodeling process, the temporary granulation tissue initially combined by FN and type III collagen is finally replaced by type I collagen (Lenselink, 2015). In addition, in the later stage of remodeling, FN plays a role by promoting the proliferation of *α*-smooth muscle actin (α-SMA) and assisting FCs to transform into myofibroblast cells (MFCs) with strong contractile effect (Sethi et al., 2002; Musiime et al., 2021; Spada et al., 2021).

However, the process of obtaining natural collagen is complex and costly. Similarly, the quantity of natural FN is limited and the price is expensive. In this study, specific functional fragments are selected by the institute of biomedicine and guangdong provincial key laboratory of bioengineering medicine of Jinan university in Guangzhou to obtain RHC and rhFN with high bioactivity and biocompatibility. RHC contains cell adhesion domains derived from natural type I collagen, which overcomes the limitations of natural animal-derived collagen. For example, the quality and purity of animal-derived collagen may affect performance, and non-human protein components may lead to an immune response in susceptible patients and a risk of contamination by pathogenic substances (Cheng et al., 2020; Deng et al., 2020). Similarly, in this study, rhFN selected fragments with fibronectin bioactivity, which overcomes the shortcomings of other methods. For instance, natural fibronectin extracted from human or animal blood and tissues is limited and expensive. At the same time, the molecular weight of wild-type fibronectin is too large to be recombined and expressed. In this experiment, we try to explore their effects on wound healing individually or in combination, and verify whether they have synergistic effect in human microenvironment.

## 2 Materials and Methods

### 2.1 Construction of RHC and rhFN

The gene encoding RHC was cloned into the plasmids containing pET-3c (Invitrogen, United States) expression vector, and the recombinant plasmid named pET3c-hlcollagen was constructed and transformed into *Escherichia coli* BL21 (DE3) (Invitrogen, United States). After that, the best expression conditions was selected by screening for ampicillin resistance and induction by isopropyl β-d-1-thiogalactopyranoside (Invitrogen, United States). A 50 L fermentor was used for generating large-scale production of RHC. In addition, RHC was purified by using affinity chromatography on a Ni Sepharose six Fast Flow (Sigma, Germany) column combined with gel filtration Sephadex G-25 (Sigma, Germany).

The gene encoding rhFN was cloned into the pPICZαA (Invitrogen, United States) expression vector to generate a recombinant plasmid named pPICZαA-rhFN. After linearizing the recombinant plasmid with sall enzyme (Takara, Japan), it was transferred into GS115 Pichia pastoris competent cells (Invitrogen, United States) and screened for positive transformants. Afterwords, recombinant human-like fibronectin was expressed by biological fermentation. Moreover, the nucleotide sequence was adapted by the host cell codon to optimize the expression of recombinant fibronectin.

### 2.2 Extraction and Identification of Dermal FCs

C57BL/6 mice were killed and their backs were depilated. Then they were washed with water and soaked in alcohol for 5 min. After that, the depilated skin on their backs was cut off and chopped with scissors as much as possible, which was then added wtih 0.2% collagenase (Sigma, Germany) and digested in the incubator for 1 h. Take out the digested skin, stop the digestion with DMEM medium, and then filter with a 70 μm cell filter, then centrifuge (1000 r, 5 min), remove the supernatant, and resuspend 10% FBS for inoculation. After the cells were stable, the primary cells were identified by immunofluorescence with 6-Diamidino-2-phenylindole dihydrochloride (DAPI) (Solarbio, China) and Fluoromount-G (SourthernBiotech, China).

### 2.3 Surface Characterization

The morphologies and structural properties of RHC and rhFN were characterized by scanning electron microscopy (Zeiss Sigma 300, Tescan, Germany). The excipients mannitol were added to RHC and rhFN, and they were frozen by gradient. The samples were placed on the table and coated with conductive carbon glue. After that, the lyophilized powder of RHC and rhFN were scanned and imaged by scanning electron microscope (SEM).

### 2.4 *In vitro* Studies

#### 2.4.1 Biocompatibility Test

C57BL/6 mice dermal FCs were used to evaluate the biocompatibility of the materials. Different concentrations of RHC and rhFN (concentration to 0.01 μM/L, 0.1 μM/L, 1 μM/L, 10 μM/L, respectively) as well as equivalent phosphate buffer saline (PBS) (gibco, United States) were completely immersed in DMEM media (gibco, United States) supplemented and 1% (v/v) penicillin/streptomycin (gibco, United States). The dermal FCs were inoculated in 96-well plates (3 × 10^3^ cells/well), and then added 100 µl of the above four media with 10% FBS (gibco, United States). Afterward, some cells in 96-well plates were culture at a specific point in time (24 h) at 37°C in a 5% CO_2_ humidified incubator. First, these cells were detected by CCK-8, the above four culture medium of these cells in 96-well plates was changed every day. After being cultured for 1, 2 and 3 days, the cultured medium was changed to a 10% CCK-8 solution and incubated for another 2.5 h at 37°C, and the absorbance of cells was recorded by a microplate reader (Multiskan Go, Thermo, United States) at the wavelength of 450 nm. According to the results of the pre-experiment, the most suitable concentration was selected and the cells were laid again. Some cells were stained by using Live/Dead cell imaging kit (Dojindo, Japan) to determine whether they were live (green) or dead (red). For the Live/Dead staining, 100 μl of the Live/Dead stock solution was added to each well. After 15 min, the cells were washed twice and observed by an inverted fluorescence microscope (DMI3000B, Leica, Germany). Another part of the cells were used for CCK-8 assay. For the CCK-8 assay, The cell viability (%) was assessed using the following formula:

$$\text{Cell}\;\text{viability}\left(\text{\%}\right)=\begin{array}{c} \left[\left(\text{OD}\text{Drug}-\text{OD}\text{Blank}\right)/\left(\text{OD}\text{Control}-\text{OD}\text{Blank}\right)\right]\\ \times 100\text{\%} \end{array}$$

#### 2.4.2 Cell Migration Assay

The effects of RHC and rhFN on cell migration were measured by the scratch assay. The dermal FCs of C57BL/6 mice were inoculated in 24-well plates (1×10^5^ cells/well) and grown to 80% to form a confluent monolayer. Cells were starved 6–8 h in FBS-free medium. Afterward, the cells were scratched with sterile pipet tips (200 μl) in the middle of the well and washed twice with PBS to mimic an incisional wound. Next, the artificial wound was covered by 1 ml FBS-free medium and was photographed by the microscope. As above, the pre-experiment of different concentrations of two recombinant proteins and equivalent PBS was carried out. The 24-well plates were replaced with the above media. Migration images were captured with a ×5 objective every 12 h for 36 h at 37°C and 5% CO_2_. According to the results of the pre-experiment, the most suitable concentration was selected and the cells were laid again to conduct a formal scratch assay. The cell migration rate (%) was calculated using the following formula:

Cell migration (%)=[(T0−Tt)/T0]×100%

where T0 is the scratch area at 0 h, and Tt is the scratch area without cell migration at different time points. Scratched wound area was quantified at each time-point using ImageJ 1.8.0 software (National Institutes of Health, United States).

#### 2.4.3 Cell Adhesion Assay

The effects of RHC and rhFN on the adhesion were determined by the crystal violet (Beyotime, China) assay. The 48-well plates were coverd with the above three optimal concentrations of media and equivalent PBS, then dermal FCs were seeded in the same plates (2×10^4^ cells/well). After 4 h, the cells were washed with PBS, fixed with 4% paraformaldehyde (PFA) (Sigma, Germany) solution for 20 min, and then washed with PBS again. After that, the cells were stained with crystal violet for 10 min and washed with PBS for 3 times. The morphology of FCs was observed by the microscope. The adhesion area rate (%) was calculated using the following formula: Adhesion area (%) = Tt/T0×100% where T0 is the total area at 0 h, and Tt is the adhesion area after 4 hours. The images were analyzed with Image-Pro Plus 6. 0 (IPP 6.0) software (National Institutes of Health, United States) to calculate the proportion of cell adhesion area.

### 2.5 *In vivo* Studies

#### 2.5.1 Establishment of Animal Model and Wound Healing Examination

Forty male C57BL/6 mice aged 6–8 weeks and weighed 25–30 g were provided from the Animal Research Centre of Guangdong (China). The animal study was reviewed and approved by the Institutional Animal Care and Use Committee (IACUC) of the General Hospital of the Southern Theatre Command (animal ethics approval number: scxk 2020–0054). Mice were anesthetized via intraperitoneal injection with 1% pentobarbital sodium solution (Sigma, Germany) (3 ml/kg), then their dorsal hairs were shaved and sterilized with iodophorthe. Each mouse was made into two pieces with the size of 1 × 1 cm round full-thickness skin defect. Three treatments were randomly applied to the wounds, while the control group was smeared with saline solution, and each wound was treated with a 0.5 ml dose of the respective treatment every day.

All wounds immediately after operation and five wounds randomly obtained from each group on days 3, 5, 7 and 10 post-surgery were harvested for wound closure analysis and morphological observation. The wounds were photographed with a Cannon digital camera (90 d, Cannon, Japan) and the wound area was measured using ImageJ software. Wound Healing Percentage (%) was determined by the following formula:

Wound healing percentage(%)=[(W0−Wt)/W0]×100%

where W0 represents the immediate postoperative wound area, and Wt represents the wound area on days 3, 5, 7 and 10, respectively.

#### 2.5.2 Histology Staining

The samples were collected and stained histologically on day 14. The excised patches, which contained the wound and normal tissue within 5 mm from the wound edge, were fixed in 4% PFA solution at 4°C over night, dehydrated with a gradient concentration of ethanol, embedded in paraffin (5 µm) and then sliced. The sections were collected and subsequently stained with haematoxylin and eosin (H&E) (Servicebio, China) and Masson (Servicebio, China) according to a manufacturer’s instructions. The images of the stained samples were taken by the microtome (RM2016, Leica, China). The thickness of the neo-epidermis was measured by ImageJ software. The condition of collagen deposition was measured by IPP software. The thickness of neo-epidermis and the mean optical density of collagen fibers were calculated by the following formula:

Epithelial thickness=epithelial area/epithelial length

Mean optical density(MOD)=integrated option density(IOD)sum/area sum

#### 2.5.3 Immunohistochemistry Staining

The paraffin sections were dewaxed and rehydrated by xylene and gradient alcohol, and then were blocked in 3% hydrogen peroxide solution for 10 min at room temperature. Next, they were stained with primary antibodies to rabbit anti-TNF-α (1:100, abcam, United Kingdom), rabbit anti-IL-1β (1:100, abcam, United Kingdom), mouse anti-IL-6 (1:100, abcam, United Kingdom), mouse anti-TGF-β1 (1:100, abcam, United Kingdom), mouse-anti VEGF (1:100, abcam, United Kingdom) and mouse anti-α-SMA (1:100, abcam, United Kingdom) and incubated overnight at 4°C. Then, the sections were incubated with goat anti-mouse IgG (1:100, abcam, United Kingdom) and goat anti-rabbit IgG (1:100, abcam, United Kingdom) at 37°C for 50 min. After being washed in PBS thrice, they were detected with 3,3′-diaminobenzidine tetrahydrochloride solution (Agilent Dako, United States) for 10 min. Eventually, the nuclei were stained with hematoxylin., and the sections were also captured by microtome. Positive results of the average value of optical density were quantified by IPP software in a blinded fashion. The expressions of various factors were calculated according to the following formula:

MOD=IOD sum/area sum

### 2.6 Statistical Analysis

SPSS 19.0 software was used to detect significant differences. All quantitative data were presented as mean ± standard deviation (SD). Statistical comparisons were analysed using one-way analysis of variance (ANOVA) and Bonferroni tests. In all figures, we used ∗ to denote *p*-values, in which ∗(*p* < 0.05), ∗∗ (*p* < 0.01), and ∗∗∗ (*p* < 0.001).

## 3 Results

### 3.1 Immunofluorescence Identification of Dermal FCs

**FIGURE 1:**
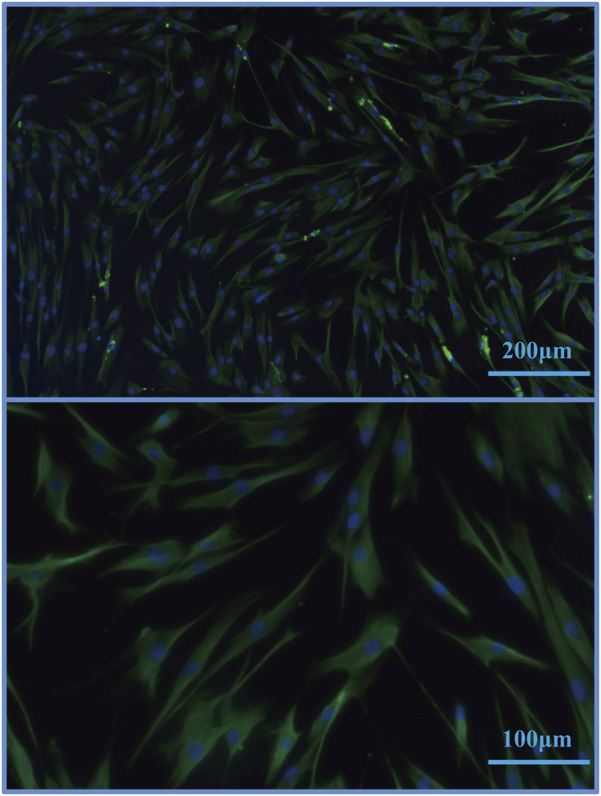
Immunofluorescence identification images of FCs.

By immunofluorescence identification, the purity of the cell reached more than 90% (Figure 1).

### 3.2 Characterization Analysis

**FIGURE 2:**
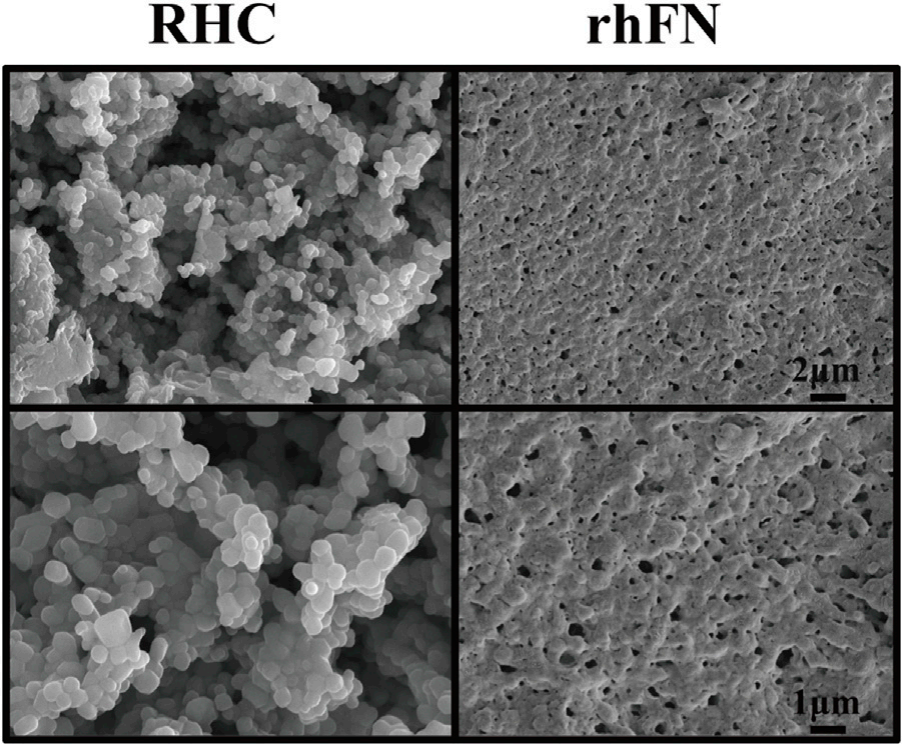
SEM images of RHC and rhFN.

Recombinant human-like collagen and recombinant human-like fibronectin were self-assemble by lyophilization to form the noticeable but irregular reticular structure, which may be used as scaffolds to produce a marked effect of chemotaxis and adhesion by attracting various cells and growth factors (Meyer, 2019) (Figure 2).

### 3.3 Enhancement of Cell Biocompatibility

**FIGURE 3:**
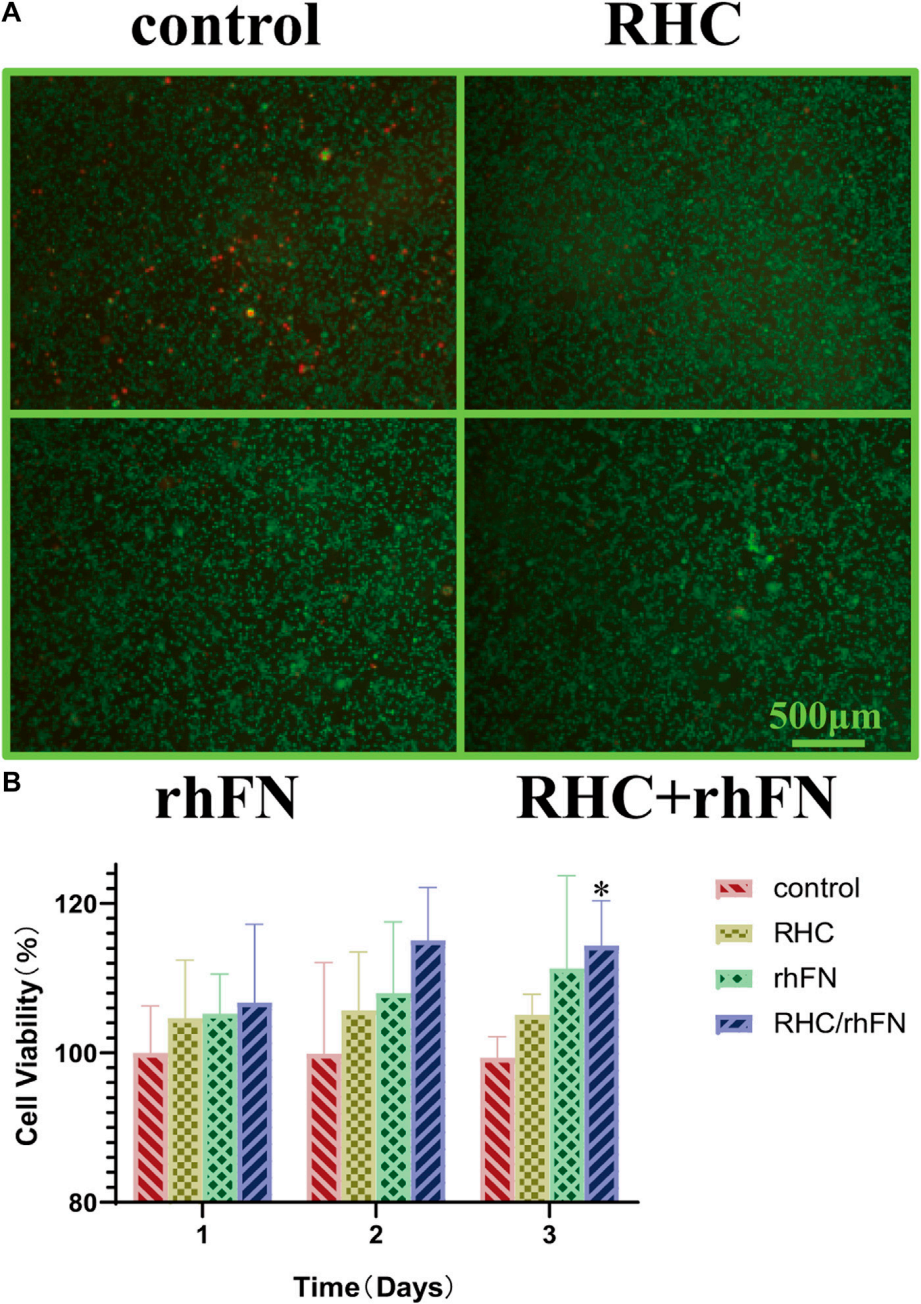
The effect of RHC + rhFN on biocompatibility with FCs. **(A)** Live/Dead staining images of FCs at hour 24. Scale bar = 500 μm **(B)** Calculation and comparison of cell viability on days 1,2 and 3. Data are presented as the mean ± SD (*n* = 6). Statistical analysis: ∗*p* < 0.05.

The biocompatibility test *in vitro* with C57BL/6 mice dermal FCs showed that RHC and rhFN had no cytotoxicity. The cells were stained with Live/Dead cell staining kit, which revealed that most of the cells were alive and almost no dead cells in all experimental groups (Figure 3A). Meanwhile, the CCK-8 assay was used to quantitatively measure the cell viability (According to the pre-experimental result, the optimal concentrations of these two recombinant proteins were both 1 μM/L). By detecting the OD value at 450 nm, all experimental groups had a higher cell viability (over 100%) compared with the control group on days 1, 2 and 3 (Figure 3B). On the third day, the cell viablity of RHC + rhFN group was significantly higher than that of the control group (*p* < 0.05), and there was no significant difference between the other groups. Both experiments revealed that RHC and rhFN were artificial biomaterials with good biocompatibility.

### 3.4 Promotion of Cell Migration

**FIGURE 4:**
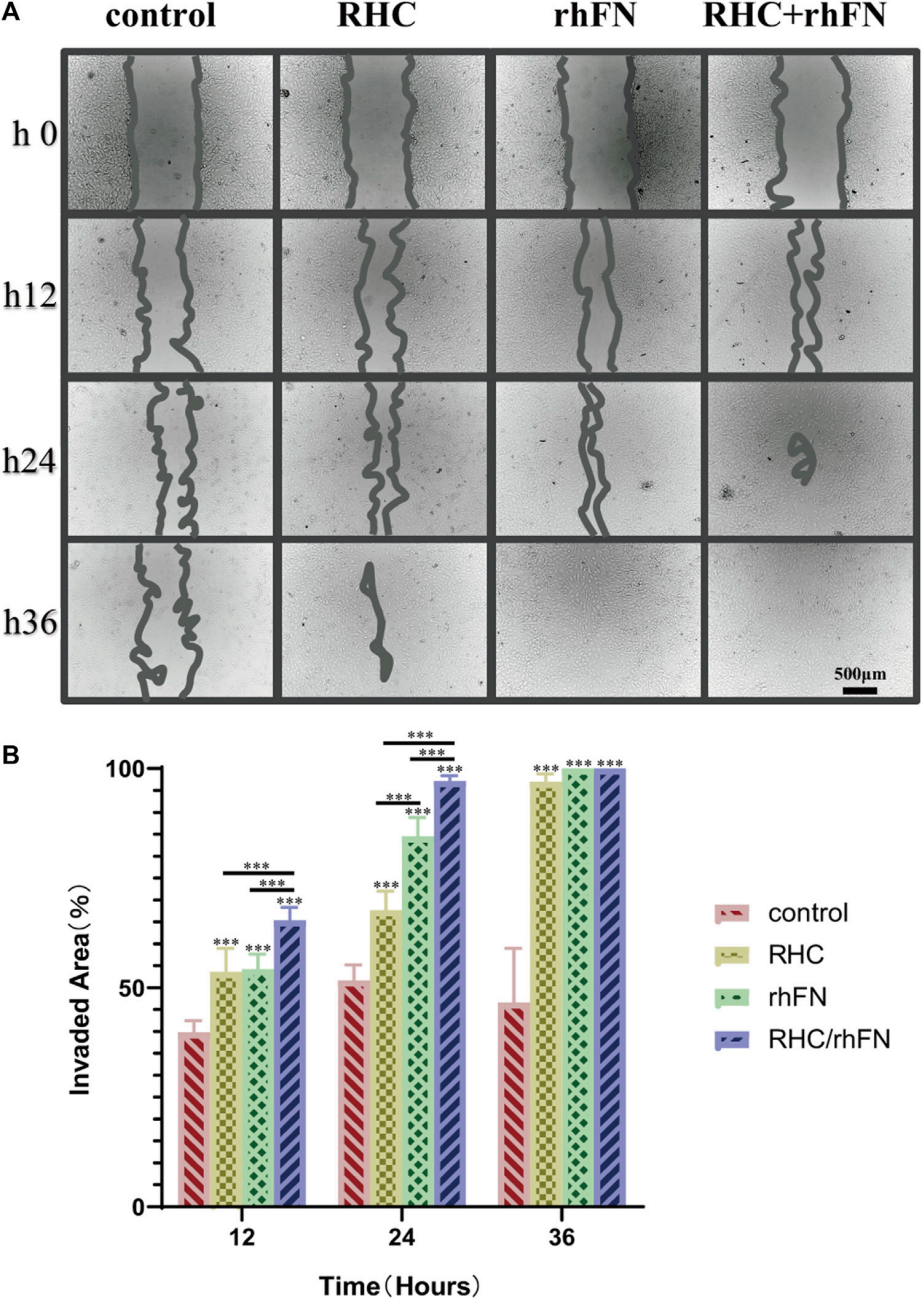
The effect of RHC + rhFN on migration with FCs. **(A)** Scratch images of FCs at hours 12, 24 and 36. Scale bar = 500 μm. **(B)** Calculation and comparison of invaded area at hours 12, 24 and 36. Data are presented as the mean ± SD (*n* = 6). Statistical analysis: ∗∗∗*p* < 0.001.

The scratch assay indicated that RHC and rhFN could promote cell migration (According to the pre-experimental result, which was proved again that the optimal concentrations were both 1 μM/L). The results showed that, in all experimental groups, wound closure by cell migration in FCs was significantly enhanced when compared with the control group, remarkably higher wound closure rates were observed in the rhFN and RHC + rhFN groups (Figures 4A,B). The faster migration in these groups was due to the fact that FN could stimulate the migration to the wound of FCs as a cell scaffold.

### 3.5 Promotion of Cell Adhesion

**FIGURE 5:**
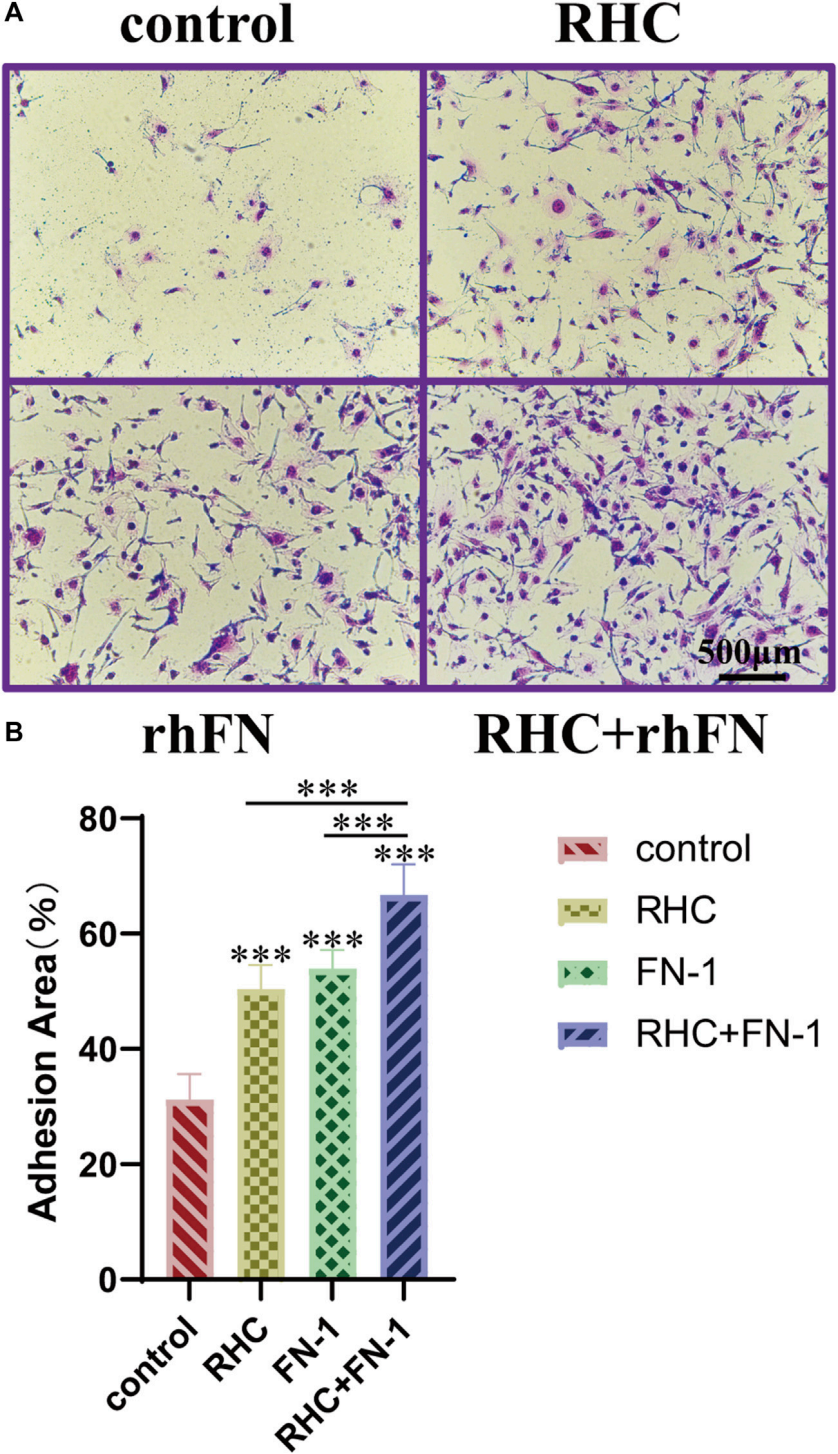
The effect of RHC + rhFN on adhesion with FCs. **(A)** Crystal violet staining images of FCs at hour 4. Scale bar = 500 μm. **(B)** Calculation and comparison of adhesion area at hour 4. Data are presented as the mean ± SD (*n* = 6). Statistical analysis: ∗∗∗*p* < 0.001.

The crystal violet assay proved that RHC and rhFN could promote cell adhesion. Compared with the control group, the experimental groups significantly improved adhesion and showed a typical spindle shape of FC (Figure 5A). In addition, we also calculated the average area proportion of attached cells. Among all the groups, the RHC + rhFN group had the highest number of attached cells that were well-spread and exhibited a typical FC morphology (Figure 5B), which may due to the synergistic effect of these two proteins.

### 3.6 The Analysis of Wound Healing Rate

**FIGURE 6:**
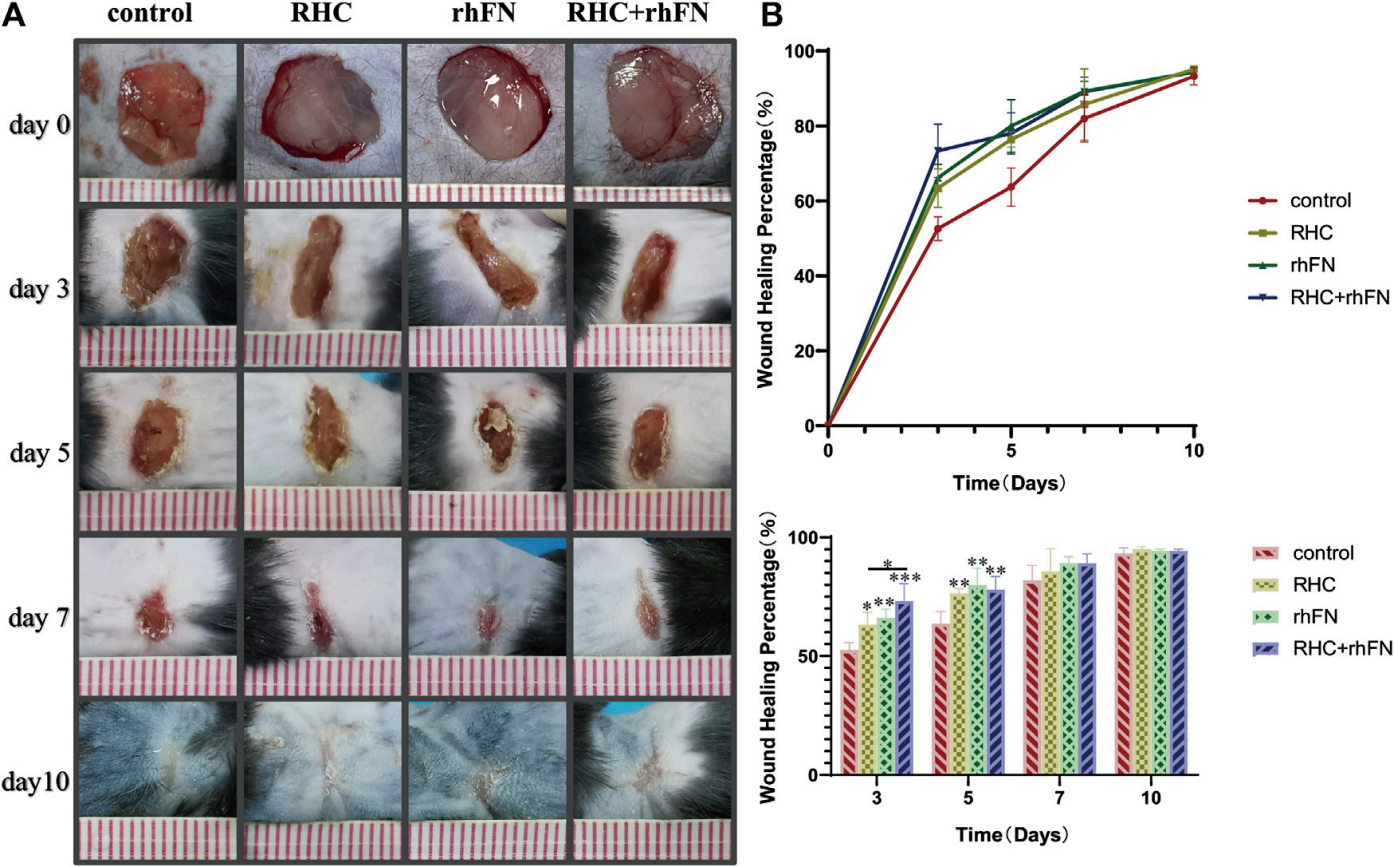
The analysis of wound healing rate. **(A)** Representative photographs on PID 3, 5, 7 and 10. **(B)** Calculation and comparison of wound healing percentage on PID 3, 5, 7 and 10. Data are presented as the mean ± SD (*n* = 5). Statistical analysis: ∗*p* < 0.05, ∗∗*p* < 0.01, ∗∗∗*p* < 0.001.

To probe into the effect of the above proteins on skin wound healing, a full-thickness skin defect model was established and the wound healing progress was analyzed at different time points after skin injury. The wound area of the model is basically same on day 0, about 1 cm^2^. On post injury day (PID) 3 and 5, there was significant difference in wound healing rates between the experimental groups and the control group. On PID 3, wound healing rates in the RHC, rhFN and RHC + rhFN groups were (63.4 ± 5.2) % (66.2 ± 3.6) % and (73.3 ± 7.1) % respectively, which were significantly higher than (52.6 ± 3.1) % in the control group. On PID 5, wound healing rates in the RHC, rhFN and RHC + rhFN groups were (76.4 ± 5.0) %, (80.0 ± 2.0) % and (78.0 ± 7.0) % respectively, which were significantly higher than (63.7 ± 5.5) % in the control group. On PID 7 and 10, wound contractions in all experimental group were similar to that in the control group. On PID 10, the wounds in all groups basically healed (Figures 6A,B).

### 3.7 The Analysis of Histology

**FIGURE 7:**
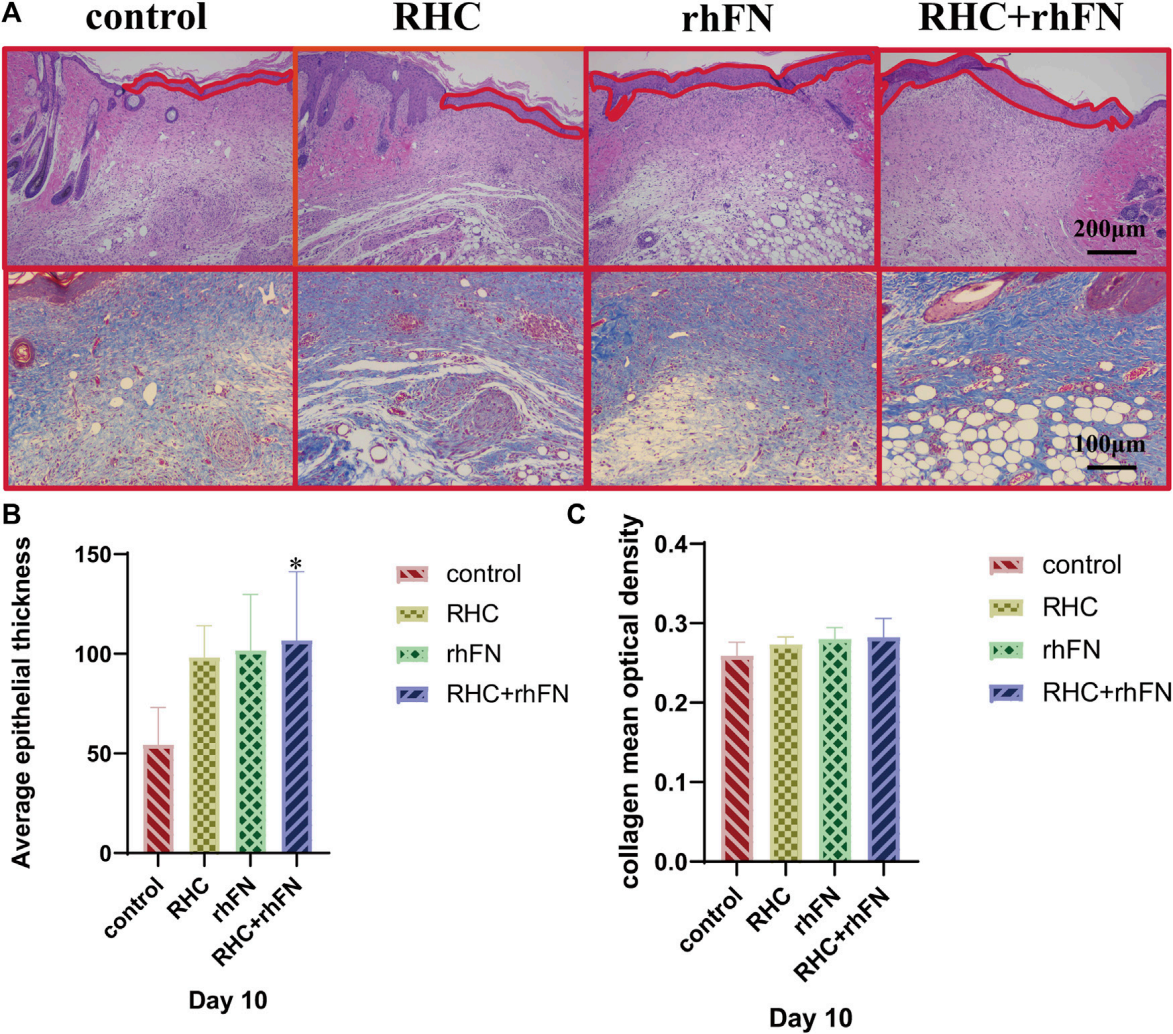
The analysis of histology. **(A)** Representative H&E staining images on PID 10. Scale bar = 200 μm. Representative Masson staining images on PID 10. Scale bar = 100 μm. **(B)** Calculation and comparison of average epithelial thickness on PID 10. Data are presented as the mean ± SD (*n* = 5). Statistical analysis: ∗*p* < 0.05. **(C)**Calculation and comparison of collagen mean optical density on PID 10. Data are presented as the mean ± SD (*n* = 5). Statistical analysis: ns *p* > 0.05.

To further investigate the quality of the newly formed skin tissue of the wound site, the wound tissues were collected on PID 10 and the morphological changes of the skin layer were observed by H&E staining. It could be seen that all groups have entered the mature stage of wound repair at this time (Figure 7A). The wound contained a large number of mature capillaries. In addtion, the complete epidermal layer was closely connected with the dermis to form the basal layer. At the same time, hair follicles, sebaceous glands and other skin accessory organs could be seen similarly (Zuijlen et al., 2003; Zheng et al., 2020). Compared with the control group, the thickness of the newly formed epidermal layers of all experimental groups were thicker, and the quantitative analysis showed that the average epithelial thickness in the RHC + rhFN group was significantly greater, while no difference was found between the other groups and the control group (Figure 7B).

Next, Masson staining was used to assess the collagen expression on PID 10 at wound site (Figure 7A). The results indicates that compared with the control group, the collagen deposition in the experimental groups had no significant difference, but arranged more regularly and orderly (Figure 7C).

### 3.8 The Analysis of Immunohistochemistry

**FIGURE 8:**
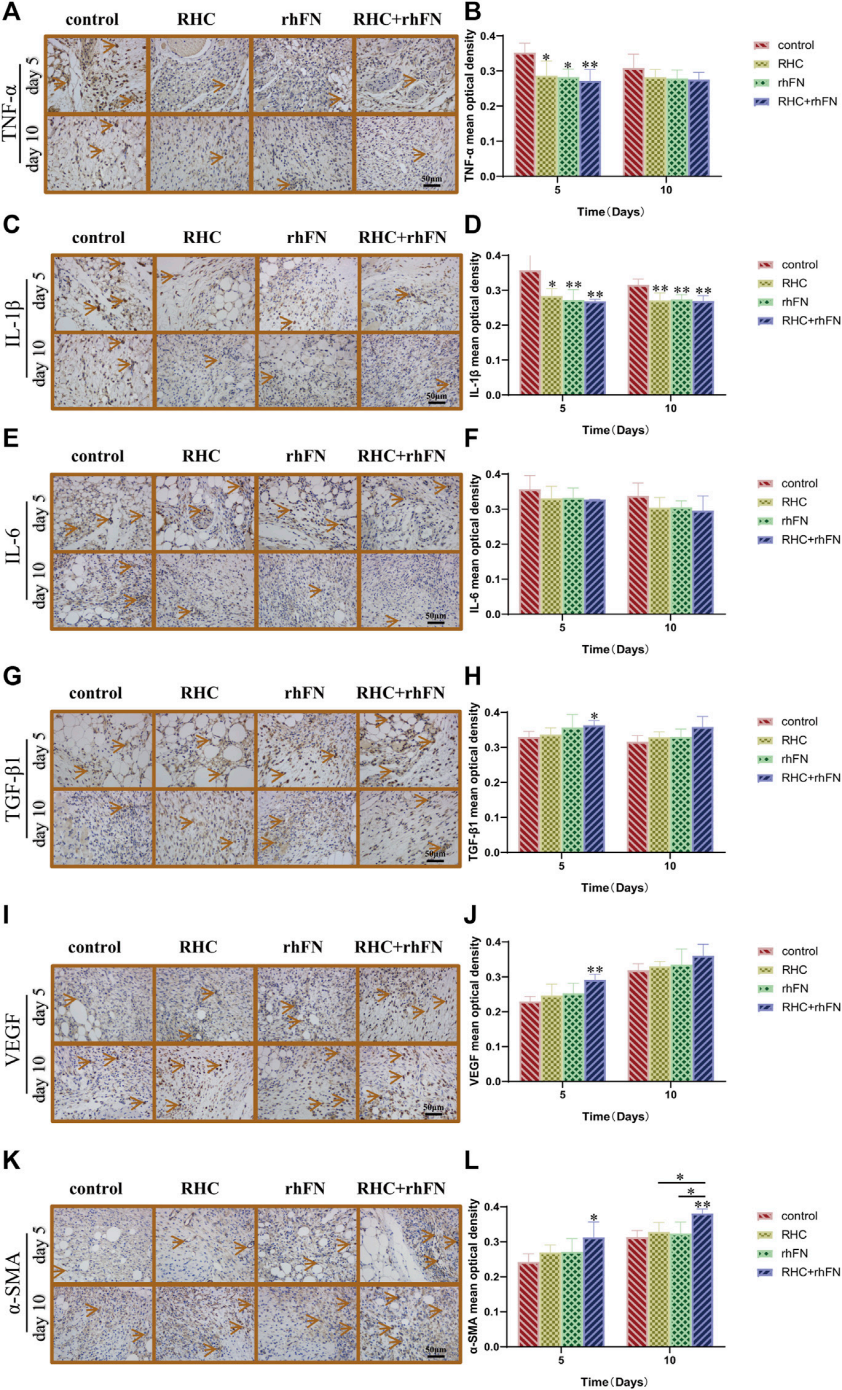
The analysis of immunohistochemistry. **(A)** Representative images of TNF-α on PID 5 and 10. Scale bar = 50 μm. **(B)** Calculation and comparison of TNF-α on PID 5 and 10. Data are presented as the mean ± SD (*n* = 5). Statistical analysis: ∗*p* < 0.05, ∗∗*p* < 0.01. **(C)** Representative images of IL -1β on PID 5 and 10. Scale bar = 50 μm. **(D)** Calculation and comparison of IL -1β on PID 5 and 10. Data are presented as the mean ± SD (*n* = 5). Statistical analysis: ∗*p* < 0.05, ∗∗*p* < 0.01. **(E)** Representative images of IL -6 on PID 5 and 10. Scale bar = 50 μm. **(F)** Calculation and comparison of IL -6 on PID 5 and 10. Data are presented as the mean ± SD (*n* = 5). Statistical analysis: ns *p* > 0.05 **(G)** Representative images of TGF-β1 on PID 5 and 10. Scale bar = 50 μm **(H)** Calculation and comparison of TGF-β1 on PID 5 and 10. Data are presented as the mean ± SD (*n* = 5). Statistical analysis: ∗*p* < 0.05 **(I)** Representative images of VEGF on PID 5 and 10. Scale bar = 50 μm **(J)** Calculation and comparison of VEGF on PID 5 and 10. Data are presented as the mean ± SD (*n* = 5). Statistical analysis: ∗∗*p* < 0.01 **(K)** Representative images of α-SMA on PID 5 and 10. Scale bar = 50 μm **(L)** Calculation and comparison of α-SMA on PID 5 and 10. Data are presented as the mean ± SD (*n* = 5). Statistical analysis: ∗*p* < 0.05, ∗∗*p* < 0.01.

Wound healing is an extremely complex inflammatory regulation process. Through immunohistochemical staining of pro-inflammatory factors (TNF-α, IL-1β and IL-6) and anti-inflammatory factor (TGF-β1), to explore the mechanism of RHC and rhFN in promoting acute wound healing in C57BL/6 mice. By the results of immunohistochemistry evaluation, on PID 5 and 10, we observed that the RHC, rhFN and RHC + rhFN groups decreased the expression of pro-inflammatory cytokines (Figures 8A–F) and increase the expression of anti-inflammatory cytokine (Figures 8G,H).

To further confirm the positive impact of RHC and rhFN on angiogenesis, we detected the expression of Vascular endothelial growth factor (VEGF) as a vascular endothelial cell marker, and α-SMA as a parietal cell marker respectively. The results proved that, compared with the control group, on PID 5, the RHC and rhFN group enhanced VEGF production (Figures 8I,J), and at the same time, α-SMA in the RHC and rhFN group was the most obvious, showing the higher vascular density on PID 5 and 10 (Figures 8K,L), both suggesting that combination therapy might have a positive impact on angiogenesis.

## 4 Discussion

Acute skin injury refers to tissue damage and continuous destruction caused by external action in a short period of time. In the process of wound healing, if it stagnates or worsens or even does not heal for a long time, it may develop into a chronic refractory wound (Ren et al., 2022). Therefore, the effective treatment of early wounds is very important to ensure the stable and orderly repair, and achieve early primary healing. At present, the clinical treatments of wound healing include a variety of interventions, such as topical dressing, biotherapy, physiotherapy and surgical treatment (Wei et al., 2021). In recent years, new bioactive materials for skin wound repair have attracted more and more attention (Hynes, 2009). In acute skin injury, collagen and FN stimulate FC formation, promote wound re-epithelialization and collagen deposition. In the meanwhile, they regulate the release of growth factors, histopathological changes, inflammatory response and angiogenesis, etc. (Manou et al., 2019). Due to poor solubility and immunogenicity, complex extraction methods and low efficiency, large molecular weight and multiple non functional domains, the application of nature protein in wound is limited. Therefore, the heterogeneity between species, the possibility of pathogen transfer and immunogenicity have prompted people to study alternative synthetic methods. Fortunately, with the progress of recombinant protein technology, the research on RHC and rhFN is increasing day by day, because of its good biocompatibility, safety, water solubility and easy production. Previous studies have successfully expressed RHC in *Escherichia coli* (Chi et al., 2011) and achieved mass production and ensured the homogeneity of different batches, as well as confirmed the effective application of recombinant collagen and derivatives combined with hydrogel in wound repair (Guo et al., 2019). Similarity, some scholars have successfully applied recombinant fibronectin and its derivatives to achieve bone repair (Xing et al., 2017; Liu et al., 2021). Consequently, these two recombinant proteins have a good prospect as a new tissue material in the field of skin repair.

According to the results of SEM, RHC and rhFN have a network structure similar to natural proteins and may act as scaffolds to attract various cells and growth factors. In previous studies, it has proved that collagen and FN can chemotactic FCs to migrate to the wound and stimulate the proliferation. Meanwhile, FCs begin to synthesize and secrete a large number of collagen fibers and matrix components to form granulation tissue to fill the wound, and converted into MFCs to promote wound contraction. In the later stage, FCs participate in tissue remodeling by secreting collagenase, and gradually apoptosis (Clark, 1993; Grinnell, 1994). We hypothesized that both recombinant proteins have binding sites of FCs and can chemotactic them, and they have synergistic effect in the process of wound repair. Combined with our experimental results *in vitro*, we have reason to believe that RHC and rhFN can promote the proliferation, migration and adhesion of FCs, and are largely interdependent and interactive, so as to jointly play the role of repair and reconstruction.

In order to further prove the effect of RHC and rhFN on wound healing *in vivo*, we used a full-thickness skin defect mouse model. In the early stage of wound repair, tissue defects are gradually filled with granulation tissue composed of new capillaries and proliferated FCs, accompanied by inflammatory cell infiltration, and its formation rate is closely related to the wound healing rate (Jiang and Scharffetter, 2020). The skin wound must undergo rapid re-epithelialization in order to achieve effective wound healing (Xu et al., 2020). The disorder of re-epithelialization is closely related to the delayed healing and the occurrence of chronic refractory wounds (Caedo-Dorantes and Caedo-Ayala, 2019). At the same time, a large amount of collagen will be generated during the remodeling period of wound healing with regularly and orderly arrangement, reducing the possibility of scar repair and improving the quality of tissue remodeling (Liu et al., 2010; Zhao et al., 2021). Our results showed that compared with the control group, RHC and rFN could significantly accelerate the speed of wound healing, especially in the combined treatment group. Besides, the combined application could increase the thickness of epithelium and improve the uniformity of collagen arrangement. Therefore, we are convinced of that RHC and rhFN play a role in the wound by promoting the early closure of the wound through a stronger degree of re-epithelialization and collagen deposition with orderly arrangement, which confirms the beneficial effect of recombinant protein on wound healing.

Inflammation is the basic response to tissue injury and invasion of pathogenic factors, as well as the inflammatory reaction initiates and regulates the process of wound repair (Li et al., 2022). Appropriate local inflammatory response can promote tissue repair, therefore, it is necessary to maintain the balance between pro-inflammatory and anti-inflammatory. According to previous studies, several pro-inflammatory cytokines play a defensive response in the early stage of wound repair by inducing inflammatory cells (Amaral et al., 2019; Huang et al., 2021). Known as is the key cytokine to initiate antibacterial inflammatory response, tumor necrosis factor-α (TNF-α) can induce activation and aggregation of inflammatory cells in the early stage. However, in the middle and late stages, too high concentration of TNF-α will cause excessive accumulation of inflammatory cells, which results in prolonged inflammatory period and poor wound healing (Ezoe and Horikoshi, 1993; Jiang et al., 2017). High concentration of interleukin-1β (IL-1β) can induce the aggravation of inflammatory reaction by activating cell cascade reaction, while low concentration of IL-1β can be combined with TNF-α to stimulate inflammatory response and promote macrophages to produce growth factors to enhance wound healing indirectly (Fedyk et al., 2001; Broekman et al., 2016; Zhou et al., 2020). Similiar, as an immunomodulatory factor, interleukin-6 (IL-6) can activate lymphocytes and promote the synthesis of reactive protein and immunoglobulin in the acute phase of repair, but down-regulate in the late stage so as to inhibit inflammation (Edara et al., 2020). Besides, transforming growth factor-β1 (TGF-β1) is a kind of multifunctional growth factor that plays an important role in wound repair (Song et al., 2002; Peters et al., 2005), which can make FCs and inflammatory cells gather to the wound site and induce granulation tissue growth, re-epithelization and collagen deposition, as well as promote the deposition of new ECM and angiogenesis (that is, granulation tissue formation) (Kishi et al., 1999). Our experiments further proved that RHC and rFN could effectively maintain tissue dynamic balance by down-regulating pro-inflammatory cytokines (TNF- α, IL-1β and IL-6) and up-regulating anti-inflammatory cytokines (TGF- β1), thus promoting skin wound repair, especially in the combined treatment group. Hence, we have a firm believe that the recombinant proteins with functional domains can produce the effect similar to natural protein. At the beginning, it produces a strong and rapid inflammatory reaction, but this reaction is temporary and subside soon, so as to promote the progress of wound healing smoothly in the next stage, and the two have synergistic effect.

Wound healing requires the participation of a variety of cells, cytokines and nutrients, and adequate oxygen supply (Eelen et al., 2020; Wei et al., 2020). As the medium of biological material transportation, blood vessel is an indispensable part in the process of wound repair. Therefore, it is necessary to restore the blood supply of damaged tissues as soon as possible and promote angiogenesis (Demidova-Rice et al., 2012; Dulmovits and Herman, 2012). By binding to the corresponding receptors on vascular endothelial cells, VEGF can strongly promote the proliferation of vascular endothelial cells and FCs in vascular adventitia, increase the permeability of neovascularization, and become the main growth factor that initiates the process of angiogenesis in granulation tissue (Werner and Grose, 2003; White et al., 2021). Vascular adventitia cells are mainly FCs after injury, FCs can be transformed into MFCs. MFCs can proliferate and migrate to neointima through the injured media, which plays an important role in the occurrence and development of vascular remodeling, and its characteristic structure is the expression of α-SMA in the cytoplasm (Sartore et al., 2001; Gingras et al., 2009; Hussain et al., 2019). In our study, the combination of RHC and rFN produced the highest expression of VEGF and α-SMA, which increased the number of neovascularization around the wound. This would determine the quality of wound healing, thus further formalizing the positive role of the combination of the two.

Based on the full-thickness skin defect mouse model, we speculate that RHC and rhFN can be widely used in wound treatment and regenerative medicine, especially in combination. Compared with the traditional extracted proteins, the new synthesized recombinant proteins have the advantages of good water solubility, easy processing, high safety, low immunogenicity, high biological activity, low cost, high preparation purity and high expression. However, due to the defects of the current technology, there are still some deficiencies. For example, most of the prepared recombinant proteins are short peptides with functional domain and lack of three-dimensional spatial structure, which makes it impossible to carve all the functions of the original protein. Moreover, they are not stable and easy to decompose and fail, so they need the specific preservation and have a short-term application. At the same time, our research also has some limitations. Firstly, the healing period of acute wound in mice is too short to observe the slight difference among RHC, rhFN and RHC + rhFN, and chronic wound and pig model may be closer to the process of human wound healing comparatively. Secondly, the prepared RHC and rhFN are in a liquid state, and they may be lost after smearing the wound, suggesting that it can be combined with hydrogel and other means to ensure the stable existence in the wound in order to perform a more effective therapeutic role. Only by solving the above problems will RHC and rhFN be of great help to our medical treatment in the future.

## 5 Conclusion

In this study, RHC and rhFN were used individually or in combination to explore the treatment of synthetic recombinant human-like collagen and recombinant human-like fibronectin on acute wounds. In this basic experiment, we verified that RHC and rhFN could improve proliferation, migration and adhesion of FCs effectively *in vitro*. In addtion, RHC and rhFN could accelerate wound healing in C57BL/6 mice and showed better re-epithelialisation and orderly structrue of collagen deposition. Moreover, RHC and rhFN could significantly maintain inflammatory balance by down-regulating pro-inflammatory factor (TNF-α,IL-1β and IL-6) and up-regulating anti-inflammatory factor (TGF-β1) as well as enhance vascularization by increasing the expression of VEGF and α-SMA. Besides, RHC and rhFN are easy to be produced on a large scale and have the clear basic mechanism of action. In conclusion, although there are still some molecular mechanisms that need to be further studied, we firmly believe that both recombinant human-like collagen and recombinant human fibronectin play a great role in wound healing and regenerative medicine.

## Data Availability

The original contributions presented in the study are included in the article/Supplementary Material, further inquiries can be directed to the corresponding author.

## Abbreviations

RHC, recombinant human-like collagen; rhFN, recombinant human-like fibronectin.
